# The National Society for Aid to the Sick and Wounded in War

**Published:** 1898-10-08

**Authors:** 


### Editor's Letter-Box.

[Our Correspondents are reminded that prolixity is a great bar to publication, and that brevity of style and conciseness of statement greatly facilitate early insertion.]

### THE NATIONAL SOCIETY FOR AID TO THE SICK AND WOUNDED IN WAR.

Major Cowen, of the Royal Army Medical Corps, writes the following account of his experience of the s.s. "Mayflower," which he took charge of for the society: "On September 6th I took over medical charge of the ship, having under my command one medical officer, one sergeant, one corporal, and six privates of the Royal Army Medical Corps. The medical and surgical equipment was supplied from the military hospital at Cairo, and proved adequate for the purpose. Three civil nurses were sent out from London by the society; they gave great satisfaction, and were zealous in the performance of their duties. The sick and wounded received military rations for the journey, which were supplemented with the society's stores or substituted where necessary. The ship was accommodated for fifty-two men; double-berthed cabins on the upper deck to accommodate thirty. On the lower deck the arrangement of the cabins has been altered, the partitions between the cabins being taken away and the berths arranged in two tiers, twelve on the starboard and ten on the port side. This proved of great convenience, as it enabled stretchers to be taken inside the cabin and right up to the berth's side. All cases confined to bed, such as wounds of the leg and dysentery, were treated, as far as accommodation permitted, on the lower deck. Assouan was reached midday of September 13th. At ten a.m. on the 15th a sick convoy, consisting of six officers and forty-four men, arrived by train from Shellal, the embarkment took place at once, and within an hour the ship left for Cairo. Of the six officers, four were wounded—three bullet and one sword—and the other two were suffering from dysentery. Of the forty-four men, twenty-seven were wounded and seventeen dysentery and fever. The wounds consisted of bullet, sword, and spear, some of the men being wounded in two and three places. The great feature in the ship, in my opinion, is that wounds could be kept at physiological rest, this being conducive to treatment and comfort not to be obtained in a railway journey. Then, again, wounds could be, and were, dressed daily on strictly antiseptic principles, thereby minimising considerably deleterious after effects.

In conclusion, I beg to express the surprise and delight of both officers and men at the ship and its comforts provided for them, and which, I feel sure, will be most gratifying to the society. The ship arrived at Cairo on the afternoon of September 18th, when the sick and wounded were conveyed by ambulances to the hospital.

